# The Association Between Experimentally Induced Stress, Performance Monitoring, and Response Inhibition: An Event-Related Potential (ERP) Analysis

**DOI:** 10.3389/fnhum.2020.00189

**Published:** 2020-06-05

**Authors:** Rebekah E. Rodeback, Ariana Hedges-Muncy, Isaac J. Hunt, Kaylie A. Carbine, Patrick R. Steffen, Michael J. Larson

**Affiliations:** ^1^Department of Psychology, Brigham Young University, Provo, UT, United States; ^2^Neuroscience Center, Brigham Young University, Provo, UT, United States

**Keywords:** event-related potential, error-related negativity, error positivity, N2, stress, Trier Social Stress Test

## Abstract

Psychological stress is increasingly associated with alterations in performance and affect. Yet, the relationship between experimentally induced psychological stress and neural indices of performance monitoring and error processing, as well as response inhibition, are unclear. Using scalp-recorded event-related potentials (ERPs), we tested the relationship between experimental stress, using the Trier Social Stress Test (TSST), and the error-related negativity (ERN), error positivity (Pe), and N2 ERP components. A final sample of 71 undergraduate students were randomly assigned to go through the TSST (*n* = 36; 18 female) or a brief mindfulness relaxation exercise (*n* = 35; 16 female) immediately followed by a go/no-go task while electroencephalogram (EEG) data were collected. Salivary cortisol, heart rate, and blood pressure confirmed increased physiological stress in the TSST group relative to control. Reaction times, accuracy, and post-error slowing did not differ by stress group. Two-group (TSST, control) by 2-trial type (correct, incorrect for ERN/Pe; go correct, no-go correct for N2) repeated measures ANOVAs for the ERN, Pe, and N2 showed the expected main effects of trial type; neither the ERN nor the N2 ERP components showed interactions with the stress manipulation. In contrast, the Pe component showed a significant Group by Trial interaction, with reduced Pe amplitude following the stress condition relative to control. Pe amplitude did not, however, correlate with cortisol reactivity. Findings suggest a reduction in Pe amplitude following experimental stress that may be associated with reduced error awareness or attention to errors following the TSST. Given the variability in the extant literature on the relationship between experimentally induced stress and neurophysiological reflections of performance monitoring, we provide another point of data and conclude that better understanding of moderating variables is needed followed by high-powered replication studies to get at the nuance that is not yet understood in the relationship between induced stress and performance monitoring/response inhibition processes.

## Introduction

Lifetime psychological stress is associated with decreased overall health and well-being (Cohen et al., 2007). For example, stress symptoms increase susceptibility to disease, cardiovascular illness, diabetes, and immune dysregulation (Maier et al., 1994; Robles and Kiecolt-Glaser, 2003). From a mental health perspective, stress symptoms can elevate the risk for depression, posttraumatic stress disorder (PTSD), anxiety disorders, and substance use (Gold and Chrousos, 2002; Howe et al., 2004; Koob and Kreek, 2007; Proulx et al., 2007). Stress also negatively affects specific cognitive processes including memory encoding and retrieval (Tollenaar et al., 2008) and attention (Bar-Haim et al., 2007; Yiend, 2010).

Performance monitoring is another cognitive process that can be influenced by stress (Compton et al., 2013). Performance monitoring is a cognitive control process that requires the ability to assess the accuracy of performance, detect errors, and adjust behavior according to one’s goals and desires (Botvinick et al., 2001). Notably, the ability to monitor performance and adjust behavior is essential in adapting to a stressful situation to accomplish the goals of the intended task (Glienke et al., 2015). For example, as individuals are engaged in a socially stressful situation, the body physiologically responds by adapting heart rate, blood pressure, and cortisol levels to meet the physiological demands the situation requires (Kelly et al., 2008; Birkett, 2011; Schoofs and Wolf, 2011). When an individual perceives the potential stressor of committing an error, the individual might subsequently adjust the amount of cognitive control used in order to complete the goal of accurate task completion and performance on subsequent trials (Glienke et al., 2015). Behavioral adaptation, therefore, occurs by the individual learning to respond to a stressor and adjust the allocation of attentional resources to successfully follow through with one’s intentions. On a broader level, neural indices of performance monitoring are moderated by situations or treatments that reduce stress and are correlated with increased life satisfaction and well-being (Robinson, 2007; Larson et al., 2010b, 2013b; Teper and Inzlicht, 2013; Whitton et al., 2017).

The neural bases of performance monitoring can be measured using multiple methods including scalp-recorded event-related potentials (ERPs) (Gehring et al., 1993, 2012; Dehaene et al., 1994; Kiehl et al., 2000; Holroyd et al., 2004; Debener et al., 2005). Imaging studies suggest a key role of the anterior cingulate cortex (ACC) in performance monitoring processes (Kiehl et al., 2000; Holroyd et al., 2004). Performance monitoring is also reflected temporally through electrophysiological signals such as the error-related negativity (ERN), correct-related negativity (CRN), and the post-error positivity (Pe) components of the scalp-recorded ERP (Larson et al., 2014).

The ERN is a response-locked, negative-going deflection in the ERP that peaks within 100 ms after committing an error (Gehring et al., 1993, 2012). The ERN is consistently related to performance monitoring as ERN amplitude is larger (i.e., more negative) when a mistake is made relative to when a correct response is provided; however, the precise functional significance of the ERN remains a matter of debate (Carter and van Veen, 2007). The cognitive processes associated with the ERN have been suggested to reflect a negative emotional response or “alarm” to affectively aversive errors (Inzlicht and Al-Khindi, 2012), a defensive response (Hajcak and Foti, 2008), the detection of competing response options (Botvinick et al., 2001; Yeung et al., 2004; Larson et al., 2014), a reinforcement learning signal (Holroyd and Coles, 2002), or early error detection (Yeung et al., 2004). Each of these theories reflect a slightly different functional significance for the ERN. However, all of these theories are associated with the idea that conflict or error processing mechanisms allow individuals to detect mistakes and adjust cognitive control to prevent future errors (Endrass et al., 2010). The ERN can be localized to the ACC (Gehring et al., 1993; Dehaene et al., 1994; Herrmann et al., 2004; Debener et al., 2005) and a convergence of information suggests the ACC plays a considerable role in self-monitoring and emotion regulation (Perrone-McGovern et al., 2017). Notably, some studies suggest a small-to-modest association between ERN amplitude and various forms of psychopathology (e.g., Pasion and Barbosa, 2019; Clayson et al., 2020).

The CRN component is the correct-trial analog of the ERN, as a smaller negative-going peak with the same timing as the ERN component (Falkenstein et al., 2000; Larson et al., 2016). The CRN may reflect just one of multiple stages of error processing (Bartholow et al., 2005), a comparison of possible responses (Vidal et al., 2000), or an emotional reaction (Luu et al., 2000), although the precise functional role of the CRN is not agreed upon.

Another error-related ERP component, the Pe (also known as the post-error positivity), is a positive-going deflection in the ERP, recorded mainly at posterior scalp sites that peaks approximately 100–400 ms after committing an error and is thought to represent conscious awareness of mistakes or attention to errors (Larson and Perlstein, 2009; Shalgi et al., 2009; Steinhauser and Yeung, 2010; Hughes and Yeung, 2011). Pe component amplitude reduces significantly when errors go undetected (Nieuwenhuis et al., 2001; O’Connell et al., 2007), but Pe amplitude may also represent the salience of an error or evaluation of the need for post-error behavior change (Hajcak et al., 2004; Forbes et al., 2008).

One final ERP component that often reflects stimulus-locked response inhibition and may be affected by stress is the N2. The N2 component is a negative-going waveform that peaks from 200 to 350 ms after the onset of a stimulus (Folstein and Van Petten, 2008). The N2 component elicited during a go/no-go task is thought to be related to inhibitory control (i.e., response inhibition), resulting in a larger (i.e., more negative) N2 amplitude to no-go trials where inhibition is required relative to go trials (Folstein and Van Petten, 2008). However, Nieuwenhuis et al. (2003) suggest that, in go/no-go tasks, the N2 may also be related to conflict monitoring (the conflict between the competing “go” and “withhold” response options) rather than reflecting purely inhibitory control processes.

Recent research suggests that neural indices of performance monitoring, particularly the ERN component, are influenced by trait indicators of stress and anxiety. For example, anxiety and stress disorders, such as PTSD and generalized anxiety disorder (GAD), are associated with altered ERN amplitudes relative to psychiatrically healthy individuals, including less negative amplitudes in individuals with PTSD (but see Swick et al., 2015), and more negative amplitudes for individuals with GAD (Hajcak et al., 2004; Clemans et al., 2012; Weinberg et al., 2012; Lieberman et al., 2017). Specific symptoms of PTSD and GAD, such as hyperarousal and high negative affect, are also associated with more negative ERN amplitudes (Hajcak et al., 2004; Lieberman et al., 2017).

As noted above, the majority of studies evaluating the relationship between the ERN component and stress or anxiety use trait indicators. Temporary (state-like) indicators of stress have thus far received less attention and have shown mixed results. For example, two studies that utilized acutely induced stressors (e.g., the presence of a tarantula near individuals with high spider fear or a cold-pressor test) had no significant effects on the ERN amplitudes of participants completing the tasks (Moser et al., 2006; Glienke et al., 2015). Alternatively, ERN amplitudes are larger (i.e., more negative) when there are large perceived negative outcomes that can be viewed as stressful or when short-term stressors build up in situations associated with occupational burnout (Hajcak et al., 2005; Golonka et al., 2017). Perhaps most relevant to the current work, a study using an analog of the Trier Social Stress Test (TSST) found that following a stressful math task, ERN amplitude was less negative compared to baseline, while the CRN amplitude was more negative, with a direct relationship to trait and state negative affect, respectively (Cavanagh and Allen, 2008). Thus, while there is a growing literature on trait-like relationships with the ERN and trait stress or anxiety, the relationship between temporary (state-like) states of stress and error-related performance monitoring is quite variable.

Similar to Cavanagh and Allen’s (2008) study, in the current study we utilized a common method for inducing social stress, the TSST. The TSST is a standard protocol for acute experimental stress tasks (Kirschbaum et al., 1993; Kudielka et al., 2007) that reliably and quickly induces psychobiological stress responses (Dickerson and Kemeny, 2004; Birkett, 2011). The TSST requires participants to prepare a speech and then present it to a research assistant, who maintains a neutral demeanor. After the speech, participants perform a stressful math task, where they are corrected immediately following an error.

Few studies that have used ERPs to examine neural responses to errors during stress (i.e., the ERN) have also examined the Pe and CRN components. The studies cited above did not include the CRN and Pe amplitudes in their results. In other work, Meyer et al. (2017) reported that the CRN is highly correlated with the ERN, and smaller CRN is related to the severity of GAD symptoms; another study found that a smaller CRN amplitude was associated with positive valence conditions (i.e., calm, happy), regardless of arousal differences (i.e., high or low; Larson et al., 2013b). The body of work on the relationship between CRN amplitude and stress states, however, is lacking.

The Pe component and its relationship to short-term stress is also unclear. As mentioned previously, Golonka et al. (2017), in their study of stressed individuals with occupation burnout, found that there was a significant decrease in Pe amplitude for the burnout group compared to the control group. A similar study on long-term (academic) stress showed that the group preparing to take a major academic exam had higher perceived stress and increased Pe amplitude than the group not preparing for an exam (Wu et al., 2014). The increased Pe amplitudes in this study may suggest that long-term academic stress allows individuals to have greater motivation to assess their errors. Cavanagh and Allen (2008) in their study of ERPs following the TSST showed that larger Pe amplitude was related to high state affect. Thus, existing evidence suggests a possible relationship between long-term or academic stress and Pe amplitude, but attention to mistakes and the functional relationship between Pe amplitude and experimentally induced stress is not known. Finally, Dierolf et al. (2018), in a relatively small sample, showed increased amplitude of the ERN and Pe components in individuals that completed the TSST, suggesting enhanced error-related processing associated with acute stress.

Reflections of inhibitory control as manifest by the N2 also suggest enhanced (i.e., more negative) amplitude during stressful conditions, whether the stress condition is a mental arithmetic task or avoiding shock (Ishida, 2006; Dierolf et al., 2017, 2018; Qi et al., 2017, 2018). The larger N2 with the stress conditions in these studies were attributed to enhanced cognitive control process, reallocation of cognitive resources to processes of inhibitory control, and a coping strategy to focus attention on threatening information. Using a modified TSST for the stress group, Jiang and Rau (2017) showed that N2 difference waves of the stress group were significantly smaller than the control group. These authors reasoned that stress impairs the response inhibition process as well as conflict detection abilities.

Given the lack of clarity about how performance monitoring is affected by stress in the extant literature, the primary purpose of the current study was to ascertain whether performance monitoring (as measured by ERN, CRN, and Pe components) and response inhibition (measured by the N2 component) are influenced by a temporarily induced social stressor (the TSST). We hypothesized that increased stress levels would be associated with increased amplitude of the ERN, the Pe, and the N2. As individuals experience increased stress and make mistakes, they will adjust cognitive resources to avoid future errors, likely reflected in larger post-error slowing in those experiencing the TSST relative to those in the non-stress condition.

## Materials and Methods

Study data, statistics code, and Supplementary Material can be found on the Open Science Framework (OSF) at https://osf.io/7a9mv/?view_only=3642a0e7202b437ba0e96ca59f5dd6be or https://tinyurl.com/s825mlo.

### Participants

Participants were recruited through undergraduate psychology classes for course credit or through flyers posted around the university campus. Exclusion criteria included current use of blood pressure or heart medications, steroid medications, psychiatric medications or diagnoses, or a history of head trauma with loss of consciousness or other neurologic conditions. Participants were compensated with course credit or $20. Upon arrival to the lab, participants were randomly assigned to either a stressful or relaxing condition using a random number generator. The original sample included 101 undergraduate students. Prior to statistics extraction, 20 participants (10 assigned to the TSST and 10 control) were excluded due to previous psychiatric diagnoses/psychotropic medication use (*n* = 9) or poor data quality such that there were no useable trials after artifact detection (*n* = 11).

Subsequently, data from the remaining 81 participants was run through the ERA Reliability Analysis (ERA) Toolbox v 0.4.4 (Clayson and Miller, 2017a, b) to determine dependability, a G-Theory analog to internal consistency reliability, using formulas provided by Baldwin et al. (2015). The ERA Toolbox used CmdStan v 2.0.1 (Stan Development Team, 2016) to implement the statistical models in Stan (Carpenter et al., 2017). As noted above, dependability is similar to internal consistency but allows for the simultaneous examination of reliability of multiple facets of the data and determination of a cut-off for a minimum number of trials to achieve adequate dependability (see Clayson and Miller, 2017b, for a full explanation and tutorial). Total numbers of participants used for analyses of each ERP component, dependability estimates, 95% credible intervals, mean number of trials, and number of trials range, separated by group, condition, and ERP components, are presented in Table 1. Ten additional participants were excluded from analyses for the two components obtained from error trials (ERN and Pe) and one from the stimulus-locked N2 component due to too few trials to reach adequate dependability on those components. As such, the final sample size for the ERN and Pe was 71 participants, while the final sample size for the N2 analyses was 80 participants. Overall demographic information and demographics broken down by TSST and control group are presented in Table 2.

**TABLE 1:** Dependability estimates for each group, condition, and ERP component.

	**Dependability**	**95% CI**	**Mean # trials**	**Trial range**
**ERN (*n* = 71)**				
TSST no-go incorrect	0.65	0.49; 0.78	26	14; 46
TSST go correct (CRN)	0.94	0.91; 0.96	214	89; 283
Control no-go incorrect	0.66	0.47; 0.80	25	13; 42
Control go correct (CRN)	0.94	0.91; 0.96	228	87; 284
**N2 (*n* = 80)**				
TSST go correct	0.96	0.94; 0.97	208	42; 283
TSST no-go correct	0.87	0.81; 0.91	42	7; 65
Control go correct	0.95	0.92; 0.97	224	72; 289
Control No-go Correct	0.79	0.69; 0.87	45	20; 63
**Pe (*n* = 71)**				
TSST no-go incorrect	0.65	0.47; 0.79	26	14; 42
TSST go correct	0.91	0.86; 0.95	227	87; 284
Control no-go incorrect	0.69	0.54; 0.81	25	11; 46
Control go correct	0.94	0.91; 0.96	208	55; 283

**TABLE 2:** Summary demographic statistics for each ERP component.

		**ERN**	**N2**	**Pe**
Total sample	*N*	71	80	71
	*N* female	34 (48%)	38 (48%)	34 (48%)
	Mean age (SD)	20.7 (2.3)	20.7 (2.3)	20.7 (2.3)
TSST condition	*N*	36	42	36
	*N* female	18 (50%)	20 (48%)	18 (50%)
	Mean age (SD)	21.2 (2.5)	21.2 (2.5)	21.2 (2.5)
Control condition	*N*	35	38	35
	*N* female	16 (46%)	18 (47%)	16 (46%)
	Mean age (SD)	20.1 (2.0)	20.1 (2.0)	20.1 (2.0)

In the absence of an *a priori* sample size calculation (see Larson and Carbine, 2017; Clayson et al., 2019), we conducted a sensitivity analysis based on the ERN/Pe sample size of 71 participants using G^∗^Power (v. 3.1.9.4; Faul et al., 2007) with an alpha of 0.05, power of 0.8, 2 groups, 2 measurements, 0.5 correlation among repeated measures, and non-sphericity correction of 1. The effect size (f) was 0.17, which indicates the current sample size and data are sensitive to detect a small-to-medium or larger effect.

### Procedure Overview

As noted above, participants were randomly assigned to task condition upon arrival. All participants provided written consent according to procedures approved by the local Institutional Review Board and in accord with the Declaration of Helsinki. Following consent, the electroencephalogram (EEG) net and blood pressure cuff were properly fitted and the participant completed a demographics survey, 10-cm visual analog scales (VAS), and the Profile of Mood States (POMS) on an iPad using Qualtrics survey software; as a note, participants also completed the State-Trait Anxiety Inventory (STAI), Outcome Questionnaire-45 (OQ-45), Beck Depression Inventory-II (BDI-II), and the Interpersonal Reactivity Index (IRI), but these questionnaires were not analyzed or reported because of lack of *a priori* hypotheses and are stated here only for completeness in method description. Participants then completed either the TSST or a relaxing mindfulness listening exercise (described below) depending on their assigned condition. Following the task, the POMS and VAS were again administered as a manipulation check. Participants then completed a go/no-go task while EEG data were recorded. After the EEG portion, participants completed a final POMS and VAS, the EEG net was removed, and the participant completed a 20-min recovery period. Throughout the participant’s time in the lab, research assistants took saliva samples and blood pressure readings from the participant at specified intervals (see sections “Salivary Cortisol and Alpha Amylase” and “Blood Pressure and Heart Rate” below for details).

#### Questionnaires

We included six VAS as a manipulation check that ranged from “not at all” to “very much,” including stressed, calm, anxious, relaxed, engaged, and bored on a 10-cm line. The calm, relaxed, and engaged VAS were reversed so higher scores reflect “worse” reports for all six VAS reports. We averaged together the stressed and calm (reversed), the anxious and relaxed (reversed), and the engaged and bored (reversed) VAS scales for a total of three outcomes. VAS have been shown to be highly reliable and consistent (Lesage et al., 2012). We used the POMS 65-item long form (subscales: Tension-Anxiety, Depression-Dejection, Anger-Hostility, Vigor-Activity, Fatigue-Inertia, Confusion-Bewilderment, and Friendliness), which has been demonstrated to be a reliable measure of mood disturbance and feelings (Cronbach’s alpha ranged from 0.82 to 0.96; Lin et al., 2014). The primary variable we gathered from the POMS is the total mood disturbance (TMD) score. The TMD score is calculated by summing the scores for the tension, depression, anger, fatigue, and confusion scores and subtracting the score for Vigor (as such, some values for the TMD score can be negative if Vigor is higher than the sum of the other scales).

#### Trier Social Stress Test

The TSST reliably induces moderate psychological stress in laboratory settings (Kirschbaum et al., 1993). In the current study, participants were told to prepare a speech for 5 min. When the 5 min were up, they were to present their speech to a designated research assistant, who was wearing a white lab coat and maintaining a neutral demeanor, and to a camera (no information was recorded on the camera). After giving a speech for 5 min, participants were asked to complete a math stressor task. Instead of the typical TSST math task, the math task in our study was the Paced Auditory Serial Addition Task (PASAT). Blood pressure and heart rate have been shown to be significantly higher during the PASAT compared to baseline or recovery periods (Mathias et al., 2004). In the PASAT, participants listen to a recording of a man saying numbers from 1 to 9. Participants mentally add the first number that they hear to the second number they hear and say the sum out loud. Then they add the second number they heard to the third number they hear and say the sum out loud. They do this over 4 trials of 50 numbers each. Each trial takes approximately 2 min, and progressively speeds up.

#### Mindfulness/Relaxation Manipulation

Participants not assigned to the stress task were assigned to a brief relaxing mindfulness practice. During this portion of the study, the participant was left alone to listen to 14:33 minutes of mindfulness recordings. The mindfulness recording was the mindfulness of breathing exercise from Jon Kabat-Zinn’s *Mindfulness for Beginners* Disk 2 CD (Kabat-Zinn, 2006; Larson et al., 2013b). This recording provides basic instruction on mindfulness meditation, encouraging active participation in focusing on attention to breathing and being mindful in the moment. Participants sat in a quiet room and listened to the recordings.

#### Go/No-Go Task

Following the TSST or relaxation listening, ERP data were recorded while all participants completed a go/no-go task. All stimuli were presented on a 17-inch computer monitor approximately 20 inches from the participants’ head. The task consisted of 5 blocks with 75 trials each where participants responded to either an M or a W in black 36-point (Arial) font on a white screen: 70% of trials were go (M), and 30% of trials were no-go (W). Each stimulus was presented for 100 ms. A fixation cross was then presented for a variable time period between 400 and 800 ms followed by the next trial. The stimuli and fixation cross were centered on the screen. Participants were asked to respond as quickly and accurately as possible to the M presented on the screen with an index finger button press of their dominant hand; they were asked to withhold their response any time a W was presented on the screen.

The go/no-go task is regularly used to elicit the ERN/CRN and Pe components of the scalp-recorded ERP. Indeed, a recent meta-analysis that included over 37 studies and 4,115 participants reported the estimated overall reliability for the ERN component using a go/no-go task (alpha = 0.75) was similar to the reliability of the ERN elicited during a flanker task (alpha = 0.69) or a Stroop task (alpha = 0.57) (Clayson, 2020), though it is important to note that reliability/internal consistency is context-dependent on each individual study and sample (Clayson and Miller, 2017a).

#### EEG Data Acquisition and Analysis

Electroencephalogram data were recorded from 128 passive Ag/AgCl passive electrodes which were equidistantly placed using a high-density geodesic sensor net and Electrical Geodesics, Inc., NA 300 amplifier system (20 K nominal gain, bandpass = 0.01–100 Hz). During data collection, EEG data were recorded continuously at 250 Hz and referenced to the vertex electrode; electrode impedances were kept at 50 kΩ or less throughout. Offline, the data were digitally filtered with 0.1 Hz first-order high-pass filter and with a 30 Hz low-pass filter (Butterworth FIR 12 db/octave filter with a 2.0 Hz roll off) in Net Station 4 software. Next, data were specifically segmented to each ERP component (see below for specific information). All windows and amplitude extraction areas were decided *a priori.*

Windows for analyses were selected based on previous research indicating the ERN peaks within 100 ms after participant response, while the Pe is a more tonic deflection that tends to be maximal between 200 and 400 ms after participant response; the stimulus-locked N2 is most negative between 200 and 400 ms after presentation of a conflict-laden stimulus such as the flanker or withholding a response in tasks such as the go/no-go (e.g., Clayson and Larson, 2011; Larson et al., 2011). We used mean amplitudes for extraction because mean amplitudes are more robust and reliable than peak measures in the presence of external noise (Clayson et al., 2013). For the response-locked ERN, CRN, and Pe, data were segmented from 200 ms before the response to 400 ms after the response and baseline adjusted from 200 to 100 ms. ERN and CRN amplitudes were extracted using a mean amplitude from 0 to 100 ms after response (Good et al., 2015). For the Pe, data were extracted using the mean amplitude from 200 to 400 ms post-response (Larson et al., 2012). For the stimulus-locked N2, we used a window from 200 ms before stimulus to 400 ms after stimulus that was baseline adjusted from −200 to 0. The mean amplitude for the N2 was calculated from 200 to 300 ms post-stimulus presentation, consistent with previous studies of stress-related changes in N2 amplitude using go/no-go tasks and acute stressors (cf., Dierolf et al., 2018).

Post-processing was completed using the ERP PCA Toolkit (Dien, 2010). Trials were marked unusable if more than 15% of channels were bad. Channels were marked bad if the difference from the minimum to maximum values within a trial epoch were more than 100 μV or if the amplitude differed from the six closest neighboring channels at some point by more than 50 μV (see Dien, 2010). Bad channels were replaced using a weighted whole-head spherical spline interpolation. Eye blinks and saccades were removed using independent components analysis (ICA) in the ERP PCA Toolkit (Dien, 2010). If components correlated at 0.9 or higher with one of two templates, one created by authors and one provided by the toolkit author, the component was removed from the data (Dien et al., 2010). Following bad channel replacement and artifact correction, data were analyzed in Dien’s Toolkit in Matlab. First, single subject averaging was completed. Then the data were average re-referenced and baseline adjusted. Data were exported to R for statistical analysis. Electrodes were chosen *a priori* based on previous studies that suggest the ERN and N2 are maximal at fronto-central electrodes and the Pe is maximal at posterior centro-parietal electrode locations (e.g., Clayson and Larson, 2011; Larson et al., 2011). For the ERN, CRN, and N2, we averaged across four frontocentral electrode sites [6 (FCz), 7, 106, and 129 (Cz)]; quantification of the Pe was done at six posterior electrode sites [54, 55 (CPz), 61, 62 (Pz), 78, and 79], in order to improve the signal-to-noise ratio for all components relative to using a single electrode (Larson et al., 2010a; Clayson and Larson, 2013; Dien, 2017).

#### Salivary Cortisol and Alpha Amylase

Participants were asked to give six salivary samples during their time in the lab, by placing a cotton swab in their mouth until saturated. The first salivary sample was initially collected at the 11th minute after the participant arrived (during baseline). The second sample was taken at the second minute of the randomized condition (beginning of either the TSST or relaxation condition), and the third at the 13th minute of the condition (end of TSST or relaxation condition). The fourth sample was collected immediately following the go/no-go task (end of EEG task). The fifth sample was gathered in the 10th minute of recovery (halfway through recovery), and the last (sixth) sample was gathered at the 20th minute of recovery (end of recovery).

Saliva samples were stored in a freezer until they were shipped for assay. Salivary cortisol was measured with a commercial immunoassay with chemiluminescence detection (CLIA; IBL, Hamburg, Germany). Alpha amylase was measured by an enzyme kinetic method. This method involved processing the saliva on a Genesis RSP8/150 liquid handling system. The saliva was diluted 1:625 with double-distilled water and then transferred into standard transparent 96-well microplates. Then, 80 ml of the alpha amylase concentrations were inserted into each well using a pipette. The microplate was heated to 37°C and then the first measurement was obtained at a wavelength of 405 nm using a standard ELIS reader. Then a second 405 nm measurement was taken after the plate was incubated for 5 min. Alpha-amylase concentrations were calculated through increases of absorbance of diluted samples (Rohleder et al., 2006). Only the first (baseline), second (beginning of randomized task; TSST or relaxation), fourth (end of go/no-go EEG task), and sixth (end of recovery) samples were sent for cortisol and alpha-amylase level analyses.

### Blood Pressure and Heart Rate

Heart rate and blood pressure measurements were collected with a properly sized and positioned cuff using the oscillometric method on a Dinamap Model 8100 automated blood pressure monitor (Critikon Corporation, Tampa, FL, United States). The cuff positioning was in accordance with the instructions from the manufacturer. Systolic blood pressure, diastolic blood pressure, and heart rate were compared using 5 averaged time periods during the study. The time period means were computed from averages of 3 measurements per participant, taken every 2 min. The 5 time periods include: (1) baseline (prior to experimental manipulation); (2) speech/control recording (during TSST speech task or while listening to the relaxing recording); (3) math/control recording (during the TSST math task or while listening to the recording); (4) go/no-go (during the EEG go/no-go task); (5) recovery.

### Statistical Analyses

#### Physiological Manipulation Checks

Cortisol values from 7 participants and alpha amylase values from 10 participants were not useable due to insufficient saliva for analysis. Cortisol and alpha amylase were analyzed using repeated measures ANOVA (4-time by 2-group) using four test swabs taken at the beginning of the baseline period, during speech prep, after the EEG task, and at the end of the recovery time. We used the Wilk–Shapiro test in R statistical software to determine the normality of the cortisol and alpha amylase samples. We then used a Box-Cox power transformation through the boxcox function (in “MASS” package) to show that the best power transformation is a logarithmic transformation of the data. All analyses of cortisol and alpha amylase were then performed on logarithmically transformed data to avoid the discrepancies that seemingly large outliers would cast on the data. Two participants are missing heart rate data and three participants are missing blood pressure data due to monitor malfunction. Heart rate, systolic blood pressure, and diastolic blood pressure measures were compared using a repeated measures ANOVA (5-time by 2-group) using means from the five averaged time periods during the study. For all physiological manipulation checks, there was a violation of sphericity for time, so all results include a Greenhouse–Geisser correction.

#### Questionnaires

The POMS data were scored using the scoring methods outlined in the manual, with a TMD value for each participant at pre- and post-manipulation used as a self-report manipulation check. Of the 80 participants with useable N2 ERP component data, 4 participants did not have complete pre- and post-POMS data and were excluded from the POMS analyses.

From the 80 participants with useable N2 ERP component data, 9.3% were missing one or more VAS ratings (56 participants had complete data). A missing value imputation random forest was subsequently used for the VAS data (missForest package in R, see code posted on the OSF page) with an imputation error estimate of 60%. The VAS data were then analyzed with calm, relaxed, and engaged scales reverse scored so all VAS data are in the same direction; higher scores on the VAS represent more negative affect. A 2-group (TSST vs. Control) by 2-time (pre vs. post) repeated measures ANOVA was used for the POMS TMD while a 2-group (TSST vs. Control) by 2-time (pre vs. post) by 3-scales (stressed, anxious, engaged) repeated measures ANOVA was used for the VAS scores. The averaged scales of the VAS scores were used to reduce Type I error (analyzing each subscale would have yielded many analyses not needed for the purpose of the manipulation check on subjective feelings). Mauchly’s test indicated a violation of sphericity for the VAS scale analyses, χ(2) = 0.82, *p* < 0.001, and the degrees of freedom were corrected accordingly using Greenhouse–Geisser corrections.

#### Behavioral Data

Of the 80 participants originally included in analyses, one had missing behavioral data due to computer malfunction in saving the behavioral file. Accuracy of performance between groups was analyzed using a 2-group (TSST vs. Control) by 2-trial (go vs. no-go) repeated measures ANOVA. We used generalized eta-squared as a measure of effect size for all ANOVA analyses. Reaction time of go-correct trials was analyzed using a 2-group independent sample *t*-test. Only go-correct trials were used because go-incorrect and no-go-correct trials do not have reaction times. We also analyzed reaction time of post-error and post-correct trials using a 2-group (TSST vs. Control) by 2-trial (post-error vs. post-correct) by 2-response type (error vs. correct) repeated measures ANOVA. Pre-error and pre-correct trials were calculated by calculating the median of go-trial correct RTs immediately preceding an error or a correct response. Post-error and post-correct RTs were calculated by taking the median of correct go trials immediately following error- and correct-trials, respectively. The difference between pre-error and post-error trials were calculated for post-error RTs. The difference between pre-correct and post-correct trials was calculated for post-correct RTs. Trials are separated by what kind of response happens prior to or after the go-correct trials, since RTs are only taken from go-correct trials (which have a response).

#### ERP Values

Correct- and error-trial ERN and Pe amplitude values were analyzed using 2-group (TSST vs. Control) by 2-trial (Correct vs. Incorrect) repeated measures ANOVAs. N2 amplitude values were analyzed using a 2-group (TSST vs. Control) by 2-trial (go correct vs. no-go correct) ANOVA. Basic assumptions of normality and kurtosis were met for these statistical analyses. As before, generalized eta squared is presented as a measure of effect size.

#### Correlations

Four correlations were performed as exploratory analyses since the Pe component showed significant between-condition stress differences: (1) cortisol data after the EEG task (Time 4) was correlated with Pe amplitude on correct trials; (2) cortisol data after recovery (Time 6) was correlated with Pe amplitude on correct trials; (3) cortisol data after the EEG task (Time 4) was correlated with Pe amplitude on error trials; (4) cortisol data after recovery (Time 6) was correlated with Pe amplitude on error trials. Cortisol data were normalized as noted above. Times 4 and 6 were used in correlations because Time 4 indicated the cortisol peak level in the TSST group, while Time 6 was the recovery period.

## Results

### Physiological Manipulation Checks

**FIGURE 1:**
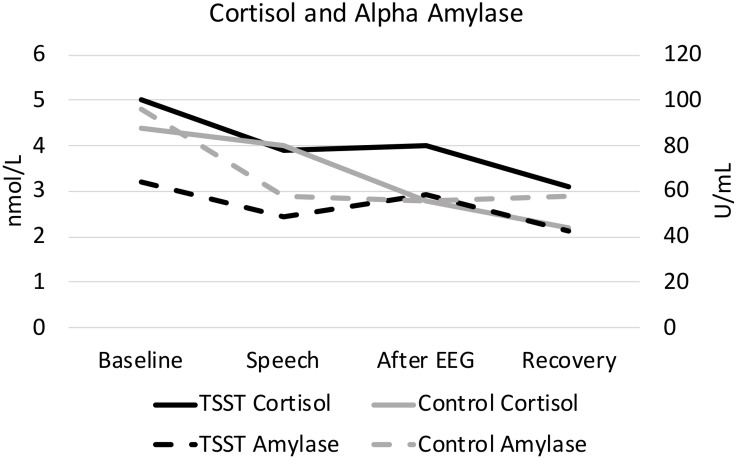
Cortisol and alpha amylase, split by group. Results are presented across the four time periods analyzed for saliva analysis (baseline, speech, after EEG, and recovery).

**FIGURE 2:**
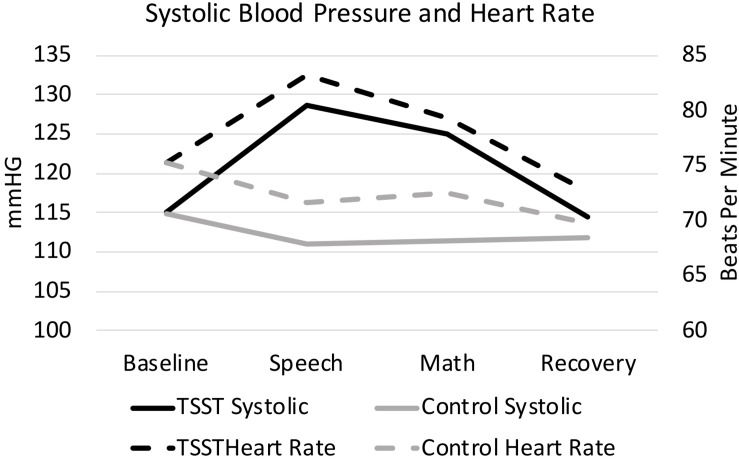
Heart rate and systolic blood pressure, split by group. Results are presented for baseline, speech, math, and recovery time periods.

As expected, the TSST was associated with increased physiological indicators of stress (see Figures 1, 2). Specifically, there was a significant interaction of group and time on salivary cortisol, while the alpha amylase only had a significant main effect of time (see Tables 3, 4). The heart rate ANOVA revealed a significant interaction of group and time, and both blood pressure ANOVAs had significant interactions of group and time as well. Notably, groups did not differ in baseline heart rate, blood pressure, or cortisol values. Thus, results suggest a difference in stress-related physiology between the TSST and control groups with the TSST group showing disproportionately increased salivary cortisol, heart rate, and blood pressure due to the stressor compared to the control participants.

**TABLE 3:** Physiological manipulation check analyses.

**ANOVA results**	**df**	**F-value**	***P*-value**	**$\eta_{\text{p}}^{2}$**
**Salivary cortisol**				
Group main effect	1, 71	1.11	0.296	0.013
Time main effect	2.07, 213	32.9	<0.001	0.068
Group × time interaction	2.07, 213	5.16	0.006	0.011
**Alpha amylase**				
Group main effect	1, 68	0.95	0.333	0.01
Time main effect	2.34, 204	6.58	<0.001	0.025
Group × time interaction	2.34, 204	1.09	0.345	0.004
**Heart rate**				
Group main effect	1, 76	3.11	0.082	0.034
Time main effect	3.36, 304	17.32	<0.001	0.032
Group × time interaction	3.36, 304	18.13	<0.001	0.033
**Blood pressure: systolic**				
Group main effect	1, 75	12.04	0.001	0.117
Time main effect	3.56, 300	22.47	<0.001	0.05
Group × time interaction	3.56, 300	46.67	<0.001	0.098
**Blood pressure: diastolic**				
Group main effect	1, 75	10.74	0.002	0.099
Time main effect	3.56, 300	20.94	<0.001	0.061
Group × time interaction	3.56, 300	45.94	<0.001	0.124

**TABLE 4:** Follow up tests on physiological manipulation check data.

		**TSST group**		**Control**			
		**Mean**	**SD**	**Mean**	**SD**	***t*-Value (between groups)**	***p*-Value**
Cortisol	Time 1	5	4.5	4.4	3.7	0.17	0.868
	Time 2	3.9	2.8	4	3.4	0.02	0.984
	Time 4	4	2.5	2.8	2	2.25	0.027
	Time 6	3.1	2.2	2.2	1.4	1.54	0.128
Alpha	Time 1	64.4	66.7	96	152.3	–1.21	0.229
amylase	Time 2	48.7	39.6	58.1	44.2	–1.16	0.252
	Time 4	58.6	45.7	55.9	49.2	0.1	0.924
	Time 6	42.3	31.8	58	54.1	–1.07	0.29
Heart rate	Baseline	75.2	12	75.2	11	–0.01	0.994
	Speech	83.2	15	71.6	11.8	3.8	<0.001
	Math	79.3	11.4	72.5	10.6	2.72	0.008
	Recovery	72.6	11.1	69.8	10.9	1.12	0.266
Systolic BP	Baseline	115.1	8.4	114.8	10.1	0.12	0.908
	Speech	128.7	12.3	110.9	9.4	7.16	<0.001
	Math	125	11.4	111.3	10.8	5.42	<0.001
	Recovery	114.4	8.6	111.8	8.9	1.31	0.193
Diastolic BP	Baseline	66.7	8.1	66.3	6.3	0.23	0.817
	Speech	77.8	9.7	63.2	6.2	7.89	<0.001
	Math	72.3	9.2	64	6.5	4.61	<0.001
	Recovery	65.5	7.2	64	5.6	0.99	0.323

*Summary statistics are on un-transformed data, while the *t* tests are on the log-transformation cortisol and alpha amylase.*

### Questionnaire Data

Visual analog scales and POMS data are presented as Supplementary Material on the OSF repository site noted above. The VAS main effect of group was not significant, *F*(1,78) = 1.73, *p* = 0.193, *$\eta_{\text{G}}^{2}=0.008$*. However, the VAS main effect of time was significant, *F*(1,78) = 53.09, *p* < 0.001, *$\eta_{\text{G}}^{2}=0.08$*, with lower VAS scores post intervention for both the TSST and control participants. The interaction between group and time was not significant, *F*(1,78) = 3.52, *p* = 0.06, *$\eta_{\text{G}}^{2}=0.005$*.

For the POMS, the main effect of group was significant, *F*(1,74) = 4.28, *p* = 0.042, *$\eta_{\text{G}}^{2}=0.05$*, with the TSST group having higher overall POMS scores. The main of effect of time was not significant, *F*(1,74) = 0.01, *p* = 0.941, *$\eta_{\text{G}}^{2}<0.001$*, nor was the interaction of group and time, *F*(1,74) = 0.5, *p* = 0.486, *$\eta_{\text{G}}^{2}=0.001$*. Thus, while there was an overall main effect of group on POMS data, the self-report measures (POMS and VAS) did not interact with the type of manipulation. The physiological measures, however, showed significant differences between groups as a function of time and task.

## Behavioral Data

Accuracy and RT data are presented in Table 5. The 2-group by 2-trial ANOVA on accuracy revealed a significant main effect of trial type, *F*(1,77) = 38.4, *p* < 0.001, *$\eta_{\text{G}}^{2}=0.25$*, with participants responding more accurately on go trials (*M* = 78% accuracy, SD = 12) than no-go trials (*M* = 64% accuracy, SD = 13). There were no significant main effects or interactions by group status for accuracy (all *p*-values > 0.05).

**TABLE 5:** Mean reaction time (milliseconds) and accuracy (percent correct) summary statistics.

	**TSST group**		**Control**	
	**Mean**	**SD**	**Mean**	**SD**
Go accuracy	78%	10	79%	14
No-go accuracy	63%	12	66%	13
Go-correct RT	202	37	205	43
Post-error on go RT	−83	67	−53	68
Post-error on no-go RT	83	94	68	96
Post-correct on go RT	18	18	16	22
Post-correct on no-go RT	−45	36	−40	46

For RTs of go-correct trials, there was a non-significant difference between groups, *t*(71.9) = −0.3, *p* = 0.7. For post-error slowing, the 2-group by 2-trial by 2-response type ANOVA on RTs revealed significant main effects of trial, *F*(1,77) = 35.4, *p* < 0.001, *$\eta_{\text{G}}^{2}=0.1$*, and response type, *F*(1,77) = 8.7, *p* = 0.004, *$\eta_{\text{G}}^{2}=0.02$*. These main effects were qualified by a significant interaction between trial and response type, *F*(1,77) = 119.5, *p* < 0.001, *$\eta_{\text{G}}^{2}=0.4$*. As expected, participants showed slower RTs after commission errors (*M* = 76 ms slowing, SD = 95 ms) than after correct go trials (*M* = 17 ms slowing, SD = 20). Participants were faster after both omission errors (*M* = 69 ms speeding, SD = 69) and “withhold” no-go correct responses (42 ms speeding, SD 41). In other words, the effect was moderated by whether or not participants responded on the error or correct trial, with faster post-response RTs after withholding and slower post-response RTs after responding. Most relevant to the aims of the current study, however, there were no significant main effects or interactions as a function of group status (all *p*-values > 0.05, see Table 5). In sum, for behavioral performance, individuals were significantly more accurate on go trials than no-go trials, but the RTs (including post-response RTs) and accuracy did not differ by stress vs. mindfulness/relaxation group.

### Event-Related Potentials

#### Error-Related Negativity

**FIGURE 3:**
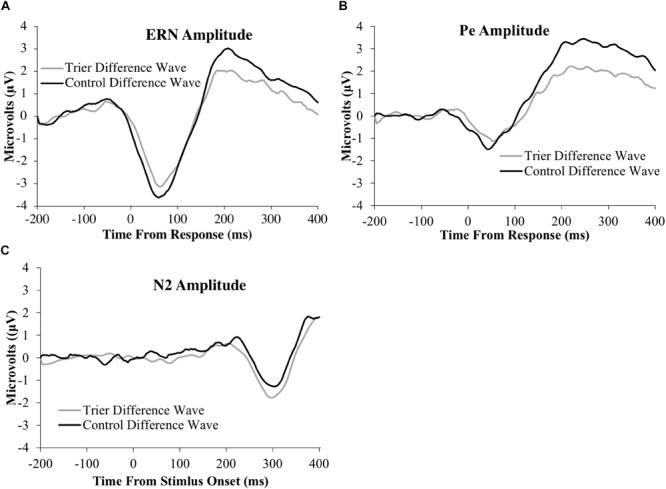
ERP difference-waves (error minus correct or no-go minus go) for the: **(A)** ERN, **(B)** Pe, and **(C)** N2 components. Data used for the difference waves for the ERN and Pe include the window from 200 ms before the response to 400 ms after the response. Data for the N2 include the window from 200 ms before stimulus presentation to 400 ms after stimulus presentation. The ERN and N2 were averaged across four frontocentral electrode sites [6 (FCz), 7,106, and 129 (Cz)]; data for the Pe were averaged across six posterior electrodes [54, 55 [CPz], 61, 62 (Pz), 78, and 79].

**FIGURE 4:**
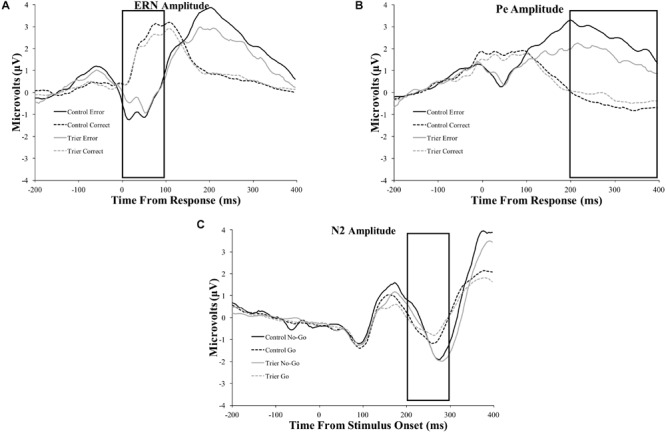
Original ERP waveforms for the: **(A)** ERN, **(B)** Pe, and **(C)** N2 components. Data for the ERN and Pe include the window from 200 ms before the response to 400 ms after the response. Data for the N2 include the window from 200 ms before stimulus presentation to 400 ms after stimulus presentation. The ERN and N2 were averaged across four frontocentral electrode sites [6 (FCz), 7, 106, and 129 (Cz)]; data for the Pe were averaged across six posterior electrodes [54, 55 (CPz), 61, 62 (Pz), 78, and 79]. Rectangles represent the time period of data extraction for each component.

All ERP amplitude values as a function of TSST and relaxation groups are presented in Table 6. Difference waves for each ERP component as a function of group status are presented in Figure 3 and the original ERPs by condition for each component are presented in Figure 4. The 2-group by 2-trial ANOVA revealed a significant main effect of trial type, *F*(1,69) = 90.3, *p* < 0.001, *$\eta_{\text{G}}^{2}=0.3$*; error trials had more negative amplitudes than correct trials (see Figure 3 and Table 6). The interaction of group and trial was not significant, *F*(1,69) = 0.7, *p* = 0.4, *$\eta_{\text{G}}^{2}=0.004$*. There was no significant main effect of group, *F*(1,69) = 0.05, *p* = 0.8, *$\eta_{\text{G}}^{2}=0.001$*.

**TABLE 6:** Mean ERP component amplitudes (μV).

	**TSST group control**			
	**Mean**	**SD**	**Mean**	**SD**
ERN: correct (CRN)	1.9	1.5	2.1	1.2
ERN: error	–0.3	2.4	–0.6	1.9
N2: correct no-go	–0.5	1.4	–0.6	1.5
N2: correct go	–1	1.8	–0.8	1.8
Pe: correct	–0.4	1.3	–0.5	1.2
Pe: error	1.6	2.6	2.9	2

#### Error Positivity

The 2-group by 2-trial ANOVA on Pe amplitude demonstrated a significant main effect of trial type, *F*(1,69) = 104.7, *p* < 0.001, *$\eta_{\text{G}}^{2}=0.4$*. Incorrect trials had more positive amplitudes than correct trials. There was no significant main effect of group, *F*(1,69) = 2.1, *p* = 0.10, *$\eta_{\text{G}}^{2}=0.02$*. Notably, there was a significant interaction of group and trial, *F*(1,69) = 7.2, *p* = 0.009, *$\eta_{\text{G}}^{2}=0.04$*. Follow-up *t*-tests indicated that only the incorrect response type differed between groups, *t*(65) = −2.2, *p* = 0.03, with the TSST group showing smaller error-trial Pe amplitude than the control group.

#### N2

The 2-group by 2-trial ANOVA on N2 amplitude showed a significant main effect of trial type, *F*(1,78) = 5.4, *p* = 0.02, *$\eta_{\text{G}}^{2}=0.009$*. No-go trials had more negative amplitudes than go trials. The main effect of group, *F*(1,78) = 0.02, *p* = 0.9, *$\eta_{\text{G}}^{2}<0.001$*, and the group by trial interaction were non-significant, *F*(1,78) = 1.1, *p* = 0.3, *$\eta_{\text{G}}^{2}=0.002$*.

To ensure that the null findings presented above were not due to the frequentist statistical methods used, we subsequently completed all of the analyses on the behavioral and ERP data using Bayesian hierarchical generalized linear models and Bayesian normal regression. The pattern of results, with a significant interaction by group for the Pe ERP, but not the ERN or N2 remained consistent. The Bayesian results were in favor of the null hypothesis for both the ERN and N2 ERPs as well as the accuracy and response time data. The full Supplementary Methods outlining the Bayesian analysis and Supplementary Results are posted on the OSF page here: https://tinyurl.com/te2wmvc.

### Correlations

Results from all four of the correlational analyses between Pe amplitude and cortisol levels were non-significant (see Table 7 for correlation values and confidence intervals).

**TABLE 7:** Correlation values between Pe component amplitude and cortisol levels.

	***r***	**95% CI**
Time 4 and correct Pe	0	−0.25; −0.25
Time 6 and correct Pe	0.02	−0.23; 0.26
Time 4 and incorrect Pe	−0.05	−0.29; 0.19
Time 6 and incorrect Pe	−0.14	−0.38; 0.11

## Discussion

Previous studies, while relatively few in number, have documented a range of results with regard to the relationship between experimentally induced stress and neural reflections of performance monitoring and response inhibition. We tested the relationship between an acute social stressor (the TSST) and the amplitude of ERP components associated with performance monitoring and response inhibition/conflict monitoring, including the ERN, Pe, and N2 components of the scalp-recorded ERP. Manipulation checks showed increased physiological indicators of stress (HR, blood pressure, and cortisol) in TSST participants relative to control participants consistent with previous work and our expectations—there were no group differences at baseline (Kirschbaum et al., 1993; Kelly et al., 2008; Birkett, 2011; Schoofs and Wolf, 2011). Notably, however, self-report measures of affect and mood (VAS and POMS TMD) did not interact with TSST or control group status. As such, while the TSST was effective in eliciting the expected blood pressure and heart rate physiological responses to stress, the participants did not report strong affective responses to the stressor, which may have influenced study outcomes or may indicate poor insight into their affective response to the stressor.

Our primary ERP finding was that of differences in Pe amplitude in individuals who were in the TSST condition compared to control participants. Error-trial Pe amplitudes were significantly smaller following the TSST than following the mindfulness/relaxation control paradigm. Pe amplitude, for either correct or error trials, however, did not correlate with cortisol levels during the stressor or after recovery. These findings provide new evidence for the relationship between error-related performance monitoring and acute stress, in an area where the extant literature is rather variable, but generally showing larger Pe-amplitudes in higher-stress individuals. For example, Wu et al. (2017) demonstrated a positive correlation between Pe amplitude and heart rate and cortisol levels following the TSST. These researchers hypothesized that the greater Pe amplitude may indicate that the more conscious the participant was of their errors, the greater stress they felt. Our findings contrast those of Wu et al. (2017); however, in relation to their theory, the smaller Pe in our sample may indicate lower awareness of errors with greater stress. One primary difference between the current study and that of Wu et al. (2017) (as well as Dierolf et al., 2017, 2018) is that their sample consisted of only males while ours had both males and females. Also, Wu et al. (2017) used a within-subjects design with a baseline measure; our study compared between subjects having two groups.

Alternatively, other work on stress associated with occupational burnout suggests smaller Pe amplitude in higher-stressed individuals (Golonka et al., 2017), consistent with our findings. Perhaps there are moderating variables that were not measured in the current study, such as punishment sensitivity (see Cavanagh and Allen, 2008, although there are outliers that influence the interpretation of their results), personality (e.g., high levels of neuroticism or conscientiousness), motivation to participate, or even the experimental task used to elicit the Pe that influence these findings (e.g., social-evaluative stressor vs. math stressor vs. occupational stressor, etc.). The specific characteristics of the tasks used to elicit errors in the various acute stress and performance monitoring studies may also have influenced the outcomes. For example, the current go/no-go task was designed to elicit errors and was very rapid (which may have been a stressor for all participants, although the cortisol, blood pressure, and heart rate data suggest not to the level of the TSST), while other similar go/no-go tasks are much slower (e.g., Dierolf et al., 2018, presented stimuli for 200 ms followed by a black screen for 1,000 ms to older adults; Dierolf et al., 2017, had the stimulus or a black screen presented until participant response followed by an interstimulus interval of 2,500 ms) or involve emotional stimuli (e.g., Jiang and Rau, 2017), or a speeded flanker task (participants had to respond within 1,000 ms) with the word WRONG presented for 500 ms after error trials (e.g., Cavanagh and Allen, 2008).

When considering the broader relationship between stress and performance monitoring/inhibition, there remains such a wide amount of variability in the tasks, degree of stress, and findings that a clear narrative is difficult to determine. For example, our findings of no differences as a function of stress group on ERN amplitude are similar to the results of Moser et al. (2006) who found no between group differences for the ERN when exposing one group (people with extreme spider fear) to a tarantula as an acutely induced stressor. However, other studies have found that stress does have an impact on ERN amplitude. For example, Cavanagh and Allen (2008) used a mathematics stressor to show baseline ERN amplitude predicted cortisol reactivity, but only in highly punishment-sensitive individuals. They interpreted their results in terms of motivation based on the role of consequences in individuals who react strongly to punishment related factors. Cavanagh and Allen (2008) were also interested in the traits associated with the stress response, while our study using the TSST is primarily focused on experimentally induced state stress. Dierolf et al. (2018) found increased ERN, Pe, and N2 amplitudes in older men who completed the TSST, suggesting a possibly adaptive role of brief stressors on error processing and inhibition. Previous research suggests that the ERN is more strongly influenced by trait personality characteristics often indicative of stress, including diagnoses of PTSD and GAD (Hajcak et al., 2004; Clemans et al., 2012; Weinberg et al., 2012; Swick et al., 2015), than state-related manipulations (e.g., Larson et al., 2013a). However, there are a range of results for state-related stress between groups, including no significant differences between stress groups (Moser et al., 2006; Glienke et al., 2015), and larger ERN amplitudes for stress-related groups (Hajcak et al., 2005; Golonka et al., 2017), as examples. Thus, potential punishment sensitivity and threat-related moderators need further examination to understand this variable area of research.

One additional possible reason for the current finding of decreased Pe amplitude in the TSST participants relative to the control participants in contrast to other studies in the literature is our use of a mindfulness-based comparison condition. We sought to maximize the difference between acute stress and acute relaxation. As such, we chose as a comparison condition a mindfulness-based paradigm (listening to the Kabat-Zinn recordings) that was easily replicable and would provide a large contrast from acute stress. We reasoned that, having participants that are acutely aware of their mistakes, such as in an acute TSST condition, compared to those that are simply mindful of their performance. Given this rationale, we anticipated increased amplitude of the ERN, Pe, and N2 components in those experiencing acute stress relative to listening to the mindfulness recordings.

Emerging literature on the relationship between performance monitoring, ERP components thought to reflect aspects of performance monitoring, and mindfulness exercises is mixed. For example, our group (Larson et al., 2013b) used the same Kabat-Zinn mindfulness recordings as those used in this study to show no significant differences between mindfulness and control groups on ERN component amplitude, but decreased Pe component amplitude in mindfulness participants. Bing-Canar et al. (2016) also used the same recordings as those used in the Larson et al. (2013b) and the current study, but found no differences between mindfulness and control groups on ERN or Pe component amplitude, although they did show heightened error-related alpha suppression in the mindfulness participants. Saunders et al. (2016) showed heightened ERN amplitude in participants who were assigned to be mindfully aware of their emotions, but not those who were assigned to be mindful of their thoughts. There was no difference between mindfulness conditions on Pe amplitude. In a randomized controlled trial with older adult participants, Smart and Segalowitz (2017) showed that participants who engaged in mindfulness activities showed increased ERN component amplitude, without a concomitant increase in Pe amplitude, compared to control participants. Finally, in the largest study to date (*n* = 212), Lin et al. (2019) tested the effects of an open monitoring mindfulness meditation on ERN and Pe component amplitudes and showed increased Pe amplitude, but no differences in ERN amplitude, compared to active control participants. Notably there was no relationship between measures of trait mindfulness and ERN or Pe component amplitude in this study. In sum, there is considerable variability on the relationship between mindfulness activities and neurophysiological measures of performance monitoring, with findings ranging from decreased Pe amplitude to increased ERN and Pe amplitude in different mindfulness interventions, with several studies showing no differences between mindfulness and control groups.

Given that we used the same mindfulness recordings and setup as previously used in our own lab where we found decreased Pe amplitude in the mindfulness participants compared to control participants, it seems unlikely that using a mindfulness control condition would account for the further decrease in Pe amplitude in the TSST participants compared to the mindfulness participants. That said, we acknowledge that using the mindfulness condition as an active control is a possible alternative explanation or, at minimum, a contributor and potential moderator for the current findings and a limitation of the current results.

The Pe and ERN results should not be considered in isolation. The N2 inhibition-related component of the ERP did not differ as a function of stress or mindfulness/relaxation group. Specifically, the N2 component did not have any significant interactions of group and trial type. The N2 results were null whereas previous research demonstrated significant results. Ishida (2006) found increased N2 amplitude with their stress group compared to the reward group, and Jiang and Rau (2017) found decreased N2 difference waves with stress compared to the control group. One possible reason for the discrepancy is variability associated with small sample sizes. Ishida (2006) had 31 people, split across two groups, and Jiang and Rau (2017) had 37 participants, split across two groups. Another reason for the difference may be differences in the degree of stress associated with the stress-induction paradigms. Ishida (2006) used a shock-avoidance task and a reward task for the different groups. Although Jiang and Rau (2017) used the TSST, their go/no-go task included emotional pictures, compared to ours that was neutral and used letters, but very fast to induce errors. These design differences, along with our use of the mindfulness/relaxation control, may account for some of the variability in N2 amplitude findings.

In addition to the ERP results, we note that the stress condition did not seem to significantly affect behavioral performance in our sample. Specifically, although the TSST was effective in increasing stress for our TSST participants, our behavioral results indicated that there was no difference in accuracy, post-error slowing rates, or RTs between groups. Taken as a whole, the majority of our current findings, with the exception of the Pe analyses, suggest that the TSST did not elicit large changes in behavioral performance or reflections of performance monitoring. It is certainly possible, then, that the Pe findings in the current study are false-positive findings. That said, our Bayesian analyses suggest an effect of TSST participation on the Pe, while the ERN and N2 results show evidence toward the null hypothesis.

One major limitation of the literature on stress and performance-monitoring/inhibition to date is that the samples used are generally quite small. For example, Wu et al. (2014) had 41 participants preparing for an academic exam (stress condition) and 20 control participants, Cavanagh and Allen (2008) had 43 participants with useable ERP data for their flanker task and 39 that complete the math stress portion of their task, and Dierolf et al. (2018) had 49 useable participants divided between two groups (25 in the stress group and 24 in the control group), Golonka et al. (2017) had 80 overall participants (40 per group), Ishida (2006) had 31 participants (18 in the stressful avoidance task and 13 in the reward task), and Jiang and Rau (2017) had 37 total participants split across the stress and control groups. The current sample is one of the largest of these studies to date with 80 useable participants for the N2 component analyses (42 in the TSST group) and 71 participants (36 in the TSST group) for the error-related ERP component analyses. The relatively small sample sizes overall in this literature may be associated with false positive or inflated findings that are prevalent throughout the neuroscience literature (e.g., Button et al., 2013). Sensitivity analyses indicate the current study was sensitive to small-to-moderate effect sizes. However, the literature is generally lacking in sample sizes and this is an area that could contribute to the variability in findings to date.

As with all studies, the current study had limitations. Dependability for each condition and ERP was somewhat lower than expected. The lowest dependability measure was 0.65 for the Pe and ERN, specifically the TSST no-go trials for the Pe and the TSST no-go incorrect for the ERN (see Table 1). The recommended dependability level is at least 0.70 (Clayson and Miller, 2017b). Thus, while the findings are not undermined by the slightly lower dependability, the dependability of the waveforms is lower than desired. Notably, however, the reliability/dependability did not differ between groups so did not likely disproportionately affect one group relative to the other. As noted above, sample size is also a possible limitation. Sensitivity analyses indicate that we were only able to detect an effect size of *f* = 0.17, which indicates sensitivity to small-to-medium effects. Also, our design included inherent differences across groups that are present in between-groups designs. We attempted to negate the effects of uneven groups through randomization of condition, and as can be seen in the demographic and mood results of the participants, the randomization seems to have led to relatively equal groups. As noted above, our groups participated in either a brief mindfulness session or the TSST. Our primary goal was to compare a stressed condition to a relaxed condition in order to more clearly examine the effects of stress on the outcome measures. Because simple control groups can have considerable heterogeneity, we used a mindfulness task to ensure participants were in a similar state during testing, but this may have enhanced the relaxation effect relative to a traditional baseline and the results should be interpreted accordingly. In addition, the TSST condition may have been associated with higher levels of physiological stress, but, as can be seen in the heart rate and systolic blood pressure data, that stress could have worn off by the end of the EEG task. Overall, the two groups may have been too similar in levels of stress by the end of the EEG task. The utilization of a go/no-go task is another possible limitation since no-go errors are compared to go correct trials (i.e., the conditions for the errors are dissimilar). However, since the primary Pe difference occurred on incorrect trials only, this is less of a concern. Finally, the speeding of the current go/no-go paradigm resulted in a useable number of errors, but it is possible that the speed of the paradigm forced a speed/accuracy tradeoff wherein participants were less cognizant of their performance and not as attentive to their mistakes, which previous research shows can influence the amplitude of the error-related ERP components (e.g., Gehring et al., 1993).

Along with limitations, this study also had several strengths. Throughout the course of the study, we directly measured the stress levels of the participants through subject ratings. We also were able to use cortisol, heart rate, and blood pressure values to demonstrate the functionality of the TSST in our study. Finally, the sample size is on the higher end of the samples to date that have examined acute stress and neurophysiological indicators of performance monitoring and inhibition. Overall, this research adds to a small amount of research that looks into how state stress affects performance monitoring ERPs. Because it is one of the first studies to explore this area of research, future studies can build on the ideas included here. Additionally, future research could include the use of an error-awareness paradigm, such as the error awareness task (EAT), to elicit the Pe during aware and unaware errors following a short-term stressor such as the TSST (Hester et al., 2005; Logan et al., 2015), and specifically test the role of punishment and threat sensitivity.

## Conclusion

In summary, current results find that the TSST was effective in inducing physiological distress in our TSST participants relative to those who engaged in brief mindfulness recordings as shown by an increase in cortisol, blood pressure, and heart rate. However, the results from the ERPs were not expected. Only the Pe had a significant interaction, with a smaller Pe following the stressor compared to the mindfulness participants. The lack of difference between the other ERPs may be due to using a state-like stress task rather than personality or punishment sensitivity variables often seen in trait-like studies of the ERN. It is possible, however, that neural processes associated with error awareness or attention to errors are influenced by induced stress as our data would suggest. The role of our mindfulness control condition is not clear and may have contributed to the current results. We conclude that there is considerable variability in the current literature on the relationship between experimentally induced stress and neurophysiological reflections of performance monitoring/inhibition. The current results provide another data point, showing reduced error-trial Pe amplitude following stress but not following a mindfulness/relaxation control, but future studies designed to moderating variables (e.g., type of stress, amount of stress, task used to elicit errors, personality variables, role of punishment and threat sensitivity, sample size, role of error awareness, etc.) are needed followed by high-powered and pre-registered studies with clear control conditions to disentangle the variability and fully understand the relationship between performance monitoring, error awareness, and acute stress.

## Data Availability Statement

Study data, statistics code, and Supplementary Material can be found on the Open Science Framework (OSF) at https://osf.io/7a9mv/?view_only=3642a0e7202b437ba0e96ca59f5dd6be or https://tinyurl.com/s825mlo.

## Ethics Statement

The studies involving human participants were reviewed and approved by the Brigham Young University Institutional Review Board. The patients/participants provided their written informed consent to participate in this study.

## Author Contributions

RR combined, analyzed, ran statistical analyses on all of the data, wrote the initial drafts and edited the manuscript. AH-M discussed best statistical procedures and helped to implement these analyses into the data, including the Bayesian analyses in the Supplementary Material, and read and edited the R code associated with these analyses and provided a second check of all statistical analyses. IH assisted with study design, IRB approval, and data collection. KC assisted with EEG analysis techniques and manuscript edits. PS helped with study design, IRB approval, analyzed cortisol and alpha amylase data, manuscript edits, and was the stress expert on this manuscript. ML oversaw and assisted all aspects of the study including study design, IRB approval, data collection, data analyses, manuscript write-up, and responses to reviewers.

## Conflict of Interest

The authors declare that the research was conducted in the absence of any commercial or financial relationships that could be construed as a potential conflict of interest.

## Supplementary Material

Study data, statistics code, and supplementary material can be found on the Open Science Framework (OSF) at https://osf.io/7a9mv/?view_only=3642a0e7202b437ba0e96ca59f5dd6be or https://tinyurl.com/s825mlo.
